# MiR-585-3p suppresses tumor proliferation and migration by directly targeting CAPN9 in high grade serous ovarian cancer

**DOI:** 10.1186/s13048-021-00841-w

**Published:** 2021-07-08

**Authors:** Xiaoyuan Lu, Guilin Li, Sicong Liu, Haihong Wang, Buze Chen

**Affiliations:** 1 Department of Gynecology, The Affiliated Hospital of Xuzhou Medical University, Jiangsu, 221000 China; 2 Department of Gynecology, Maternal and Child Health Care Hospital Affiliated to Xuzhou Medical University, Jiangsu, 221000 China; 3 Graduate School, Xuzhou Medical University, Jiangsu, 221000 China; 4 Xuzhou Medical University, Jiangsu, 221000 China

**Keywords:** Ovarian cancer, miR-585-3p, CAPN9, Bioinformatics, Cell biology

## Abstract

**Background:**

Aberrant expression of microRNAs (miRNAs) contributes to the development of high grade serous ovarian cancer (HGSOC). However, the molecular mechanism by which miRNA-585-3p mediates high-grade serous ovarian carcinogenesis is unclear. This study aims to investigate the specific mechanism of action of miR-585-3p in HGSOC.

**Methods:**

Expression of miR-585-3p in HGSOC tissues and cell lines was detected by qRT-PCR. Cell viability and migration were detected using MTT and transwell system. The expression of target genes and target proteins of miR-585-3p was detected by dual luciferase reporter assay and western blot.

**Results:**

The expression of miR-585-3p was significantly lower in HGSOC tissues and cells than in normal ovarian tissues and cell lines. In HGSOC tissues, CAPN9 expression was inversely correlated with miR-585-3p expression. MiR-585-3p inhibited the proliferation and migration of HGSOC cells. MiR-585-3p bound to the 3'-untranslated region (UTR) of CAPN9 and inhibits CAPN9 expression. Overexpression of CAPN9 reduced the inhibitory effect of miR-585-3p in HGSOC cells.

**Conclusions:**

miR-585-3p is significantly down-regulated in HGSOC tissues and cell lines. MiR-585-3p inhibits the proliferation and migration of HGSOC cells by targeting CAPN9.

**Supplementary Information:**

The online version contains supplementary material available at 10.1186/s13048-021-00841-w.

## Introduction

In worldwide, there are only an estimated 239,000 new cases of ovarian cancer (OC) and 152,000 deaths each year [1]. OC has the highest mortality rate of all gynecologic tumors, which is one of the main causes of death from gynecologic malignancies in China [2, 3]. OC exhibits a wide range of clinical, histopathological, and molecular heterogeneity and has been put into several subtypes, about 70% of which are high grade serous ovarian cancer (HGSOC) subtypes [4]. 80% of patients with HGSOC are diagnosed at an advanced stage for reasons including non-specific clinical symptoms and lack of reliable early detection biomarkers [3]. Development of targeted therapies to identify new molecular targets is necessary [5]. Therefore, it is important to investigate the mechanisms of ovarian carcinogenesis to improve the outcomes of patients with HGSOC.

MiRNAs play an important role in a variety of biological processes including cell proliferation, apoptosis, differentiation, and migration of tumors [6]. MiRNAs negatively regulate gene expression by inhibiting the translation of messenger RNA (mRNA) or by degrading mRNA [7]. The role of miR-585-3p has been explored in some cancers. Downregulated miR-585-3p promotes cell growth and proliferation in colon cancer by unregulated PSME3 [8]. MicroRNA-585 inhibits human glioma cell proliferation by directly targeting MDM2 [9]. MicroRNA-585, directly targeting MAPK1, suppresses gastric tumor proliferation and migration in gastric cancer [10]. These suggested that miR-585-3p may play an important role in HGSOC.

In this study, the expression levels and biological functions of miRNA-585-3p and its target genes in HGSOC were explored by bioinformatics and molecular biology methods. This study provides a theoretical basis for the treatment of HGSOC.

## Materials and methods

### Samples and Cell lines

Samples of tumor tissues and normal ovarian tissues from 10 HGSOC patients were collected at the Department of Obstetrics and Gynaecology of the Affiliated Hospital of Xuzhou Medical University. This study was approved by the ethics committee of Xuzhou Medical University Affiliated Hospital. All patients signed a written informed consent form. As shown in Table S1, the clinical characteristics of ten HGSOC patients were listed.

The OC cell lines SKOV3 and A2780, and the human ovarian surface epithelial cell line IOSE29, preserved in our laboratory, were used in this study. The cells were cultured with reference to the literature [5].

### QRT-PCR

The miR-585-3p levels in 10 HGSOC tissue samples and the SKOV3, A2780 and IOSE29 cell lines were identified by qRT-PCR. The detailed steps were carried out according to the literature [5]. The primer sequences used were shown in Table S2.

### Plasmid construction and transfection

MiR-585-3p mimics, miR-585-3p antisenses oligonucleotides (ASO) and control gene were designed and purchased from Sangon Biotech Company (https://www.sangon.com/; Shanghai, China). The sequences were listed in Table S2. Lipofectamine® 2000 reagent (Thermo Fisher Scientific, Waltham, MA, USA) was used to transport miRNAs into the cells. The effect of CAPN9 overexpression on the proliferation of SKOV3 cell lines was studied using the pcDNA3.1 vector.

### Cell viability and migration

Cell viability was checked by MTT assay [11]. Cell migration is detected by the Transwell system [12]. The detailed steps were carried out according to the literature [5].

### The targets of miR-585-3p

TargetScan database (http://www.targetscan.org) and miRDB database (http://mirdb.org/) were used to predict putative targets of miR-585-3p [13–16].

### Analysis of miR-585-3p and CAPN9 interactions

The detailed steps of dual luciferase reporter and western blot were carried out according to the literature [5]. CAPN9 3'-UTR sentinel mutagenesis using the Site-Directed Mutagenesis kit (Invitrogen, USA). For CAPN9 protein expression, an anti-CAPN9 antibody (Cat. No, PA5-54,197, Invitrogen, Carlsbad, CA, USA) was used at a dilution of 1:1000.

### Statistical analyses

All calculations were performed using GraphPad Prism 7.0 software. Analyses between the two groups were compared using a two-tailed Student's t-test. Analyses between the three groups were conducted using one-way ANOVA with Student–Newman–Keuls test for post hoc comparisons. P < 0.05 indicated statistical significance.

## Results

### miR-585-3p was decreased in HGSOC tissues

**Fig. 1:**
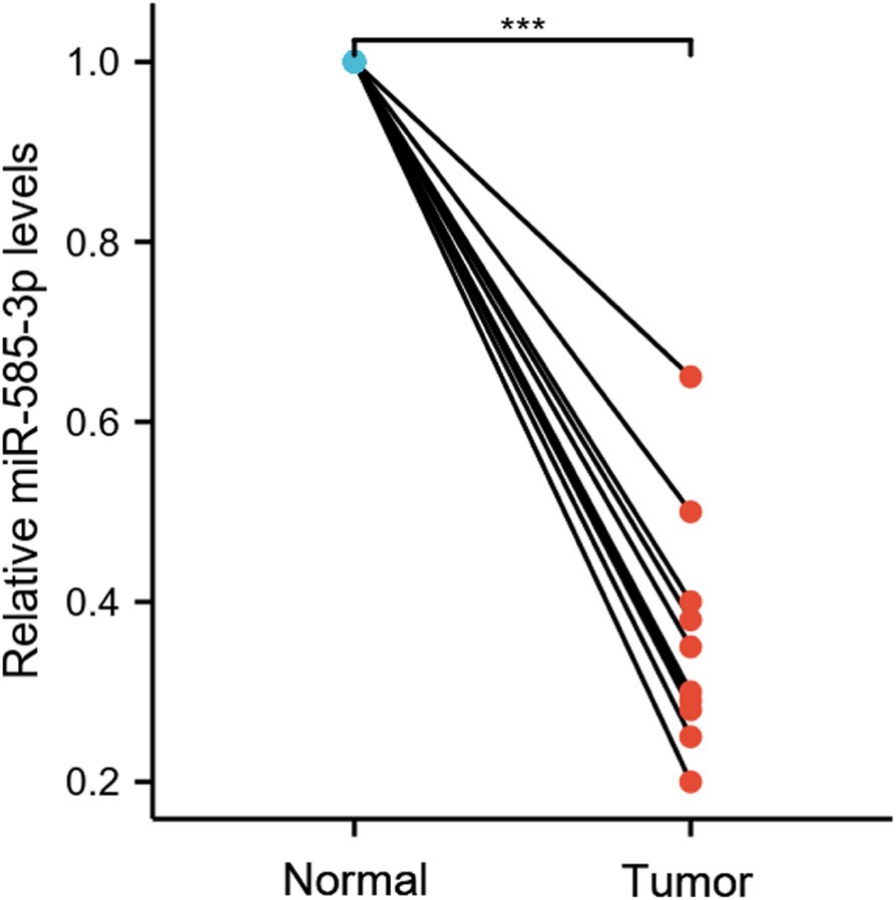
The expression of miR-585-3p was significantly lower in HGSOC tissue samples than in adjacent normal ovarian tissues. *** P < 0.001

As shown in Fig. 1, MiR-585-3p levels in tumor tissues were significantly lower than those in adjacent normal ovarian tissues (1.00 ± 0.000 vs 0.36 ± 0.042, P < 0.001).

### High expression of miR-585-3p inhibited cell viability and migration

**Fig. 2:**
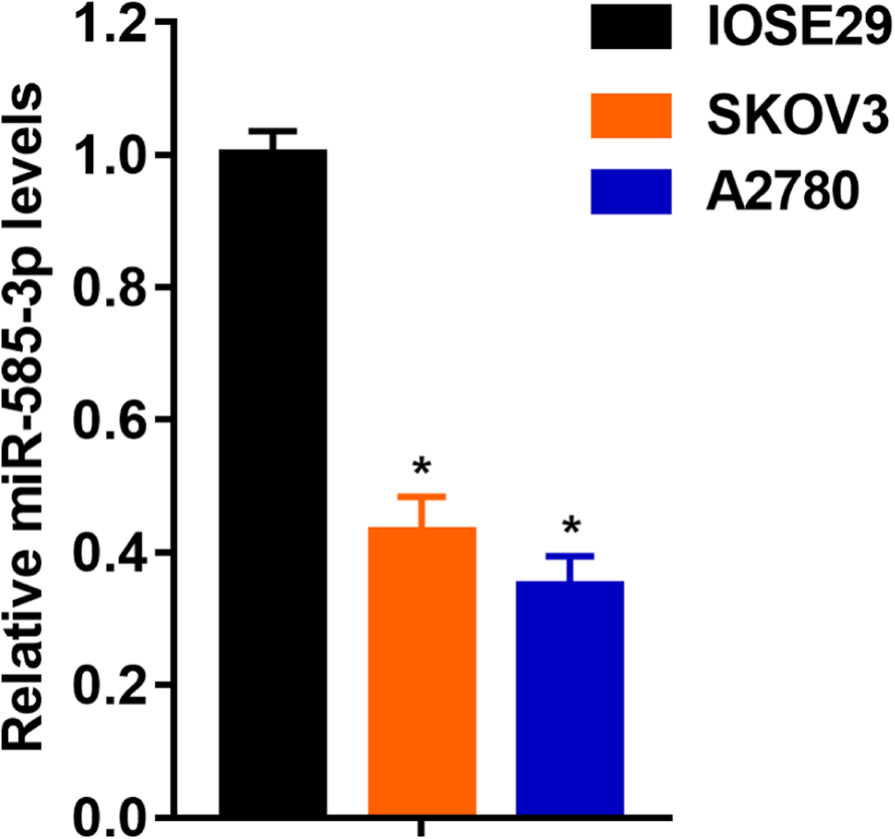
Expression of miR-585-3p in human normal ovarian epithelial cells, ovarian cancer cell lines SKOV3 and A2780

**Fig. 3:**
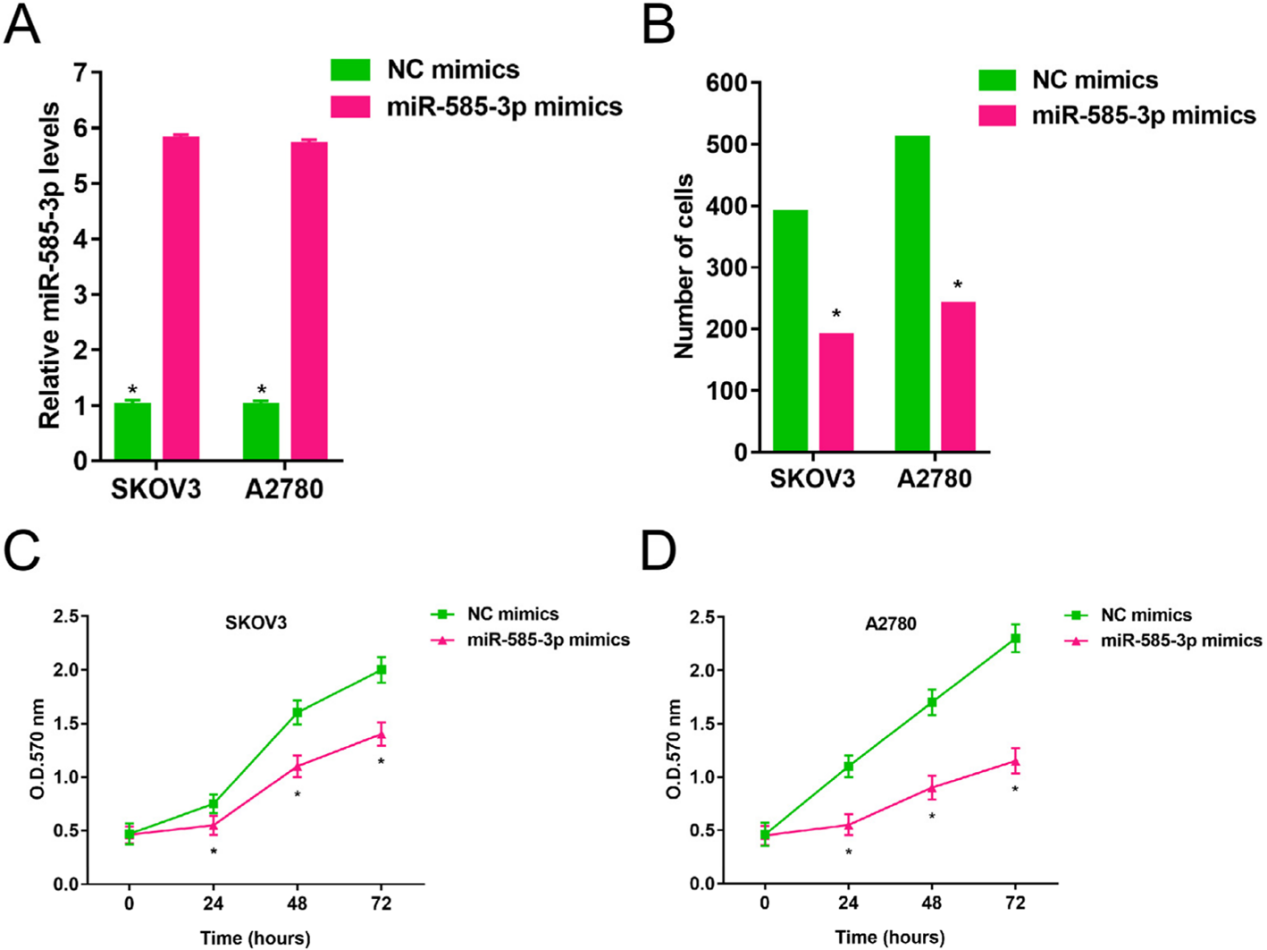
Overexpression of miR-585-3p inhibited proliferation and migration of SKOV3 and A2780. **A** Expression of miR-585-3p in transfected cells. **B** Assessment of migration of transfected cells. **C** Cell growth of SKOV3. **D** Cell growth of A2780. *P < 0.05

As shown in Fig. 2, SKOV3 and A2780 cells showed significantly lower levels of miR-585-3p compared to IOSE29 cells (1.00 ± 0.036 vs 0.043 ± 0.05, P < 0.05; 1.00 ± 0.036 vs 0.35 ± 0.05, P < 0.05). As shown in Fig. 3A, miR-585-3p mimics increased levels of miR-585-3p in SKOV3 and A2780 cells (1.00 ± 0.10 vs 5.80 ± 0.08, P < 0.05; 1.00 ± 0.08 vs 5.70 ± 0.09, P < 0.05). As shown in Fig. 3B, the viability of SKOV3 and A2780 cells was significantly inhibited within 72 h after transfection with miR-585-3p mimics (390 ± 0.5 vs 190 ± 0.6, P < 0.05; 510 ± 0.4 vs 230 ± 0.4, P < 0.05). As shown in Fig. 3C and Fig. 3D, the number of migrating cells was significantly inhibited by miR-585-3p mimics.

### Low expression of miR-585-3p promoted cell viability and migration

**Fig. 4:**
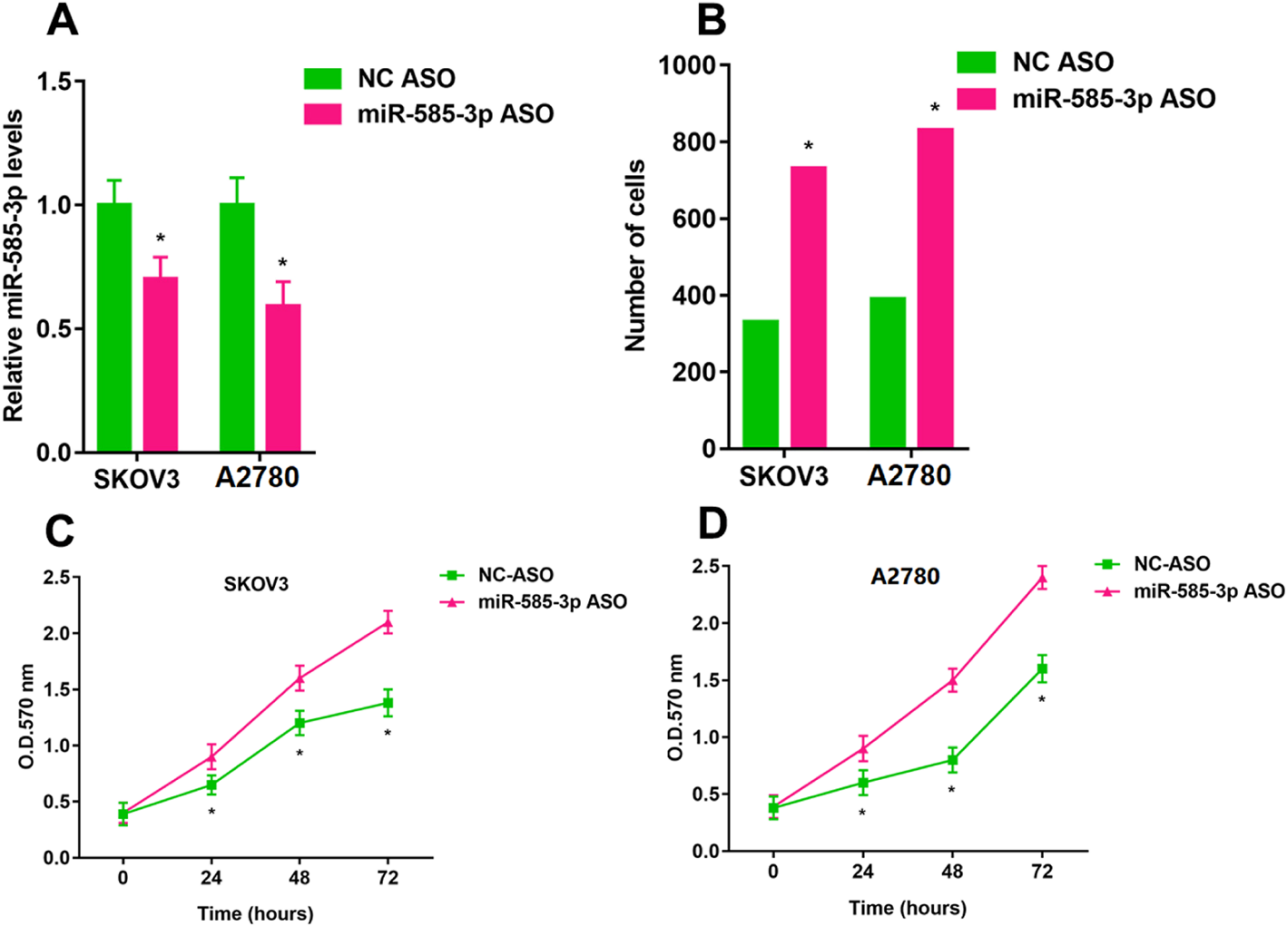
Downregulation of miR-585-3p promotes proliferation and migration of SKOV3 and A2780. **A** Expression of miR-585-3p in transfected cells. **B** Assessment of migration of transfected cells. **C** Cell growth of SKOV3. **D** Cell growth of A2780. *P < 0.05

As shown in Fig. 4A, the miR-585-3p ASO inhibited levels of miR-585-3p in SKOV3 and A2780 cells (1.00 ± 0.10 vs 0.70 ± 0.09, P < 0.05; 1.00 ± 0.11 vs 0.59 ± 0.10, P < 0.05). The viability of SKOV3 and A2780 cells was significantly promoted within 72 h after transfection with miR-585-3p ASO (330 ± 0.12 vs 730 ± 0.10, P < 0.05; 390 ± 0.13 vs 830 ± 0.12, P < 0.05) (Fig. 4B). miR-585-3p ASO inhibited the number of migrating cells (Fig. 4C and Fig. 4D).

### miR-585-3p directly targeted the CAPN9

**Fig. 5:**
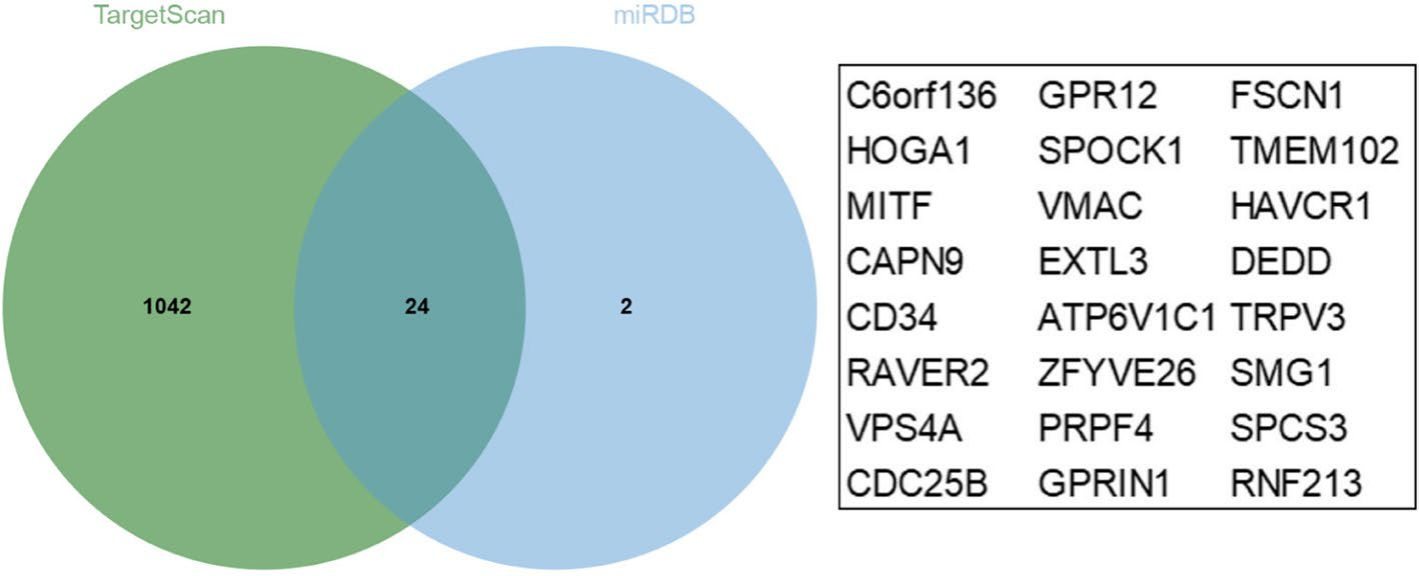
Common genes for miR-585-3p target genes predicted by TargetScan and miRDB

**Fig. 6:**
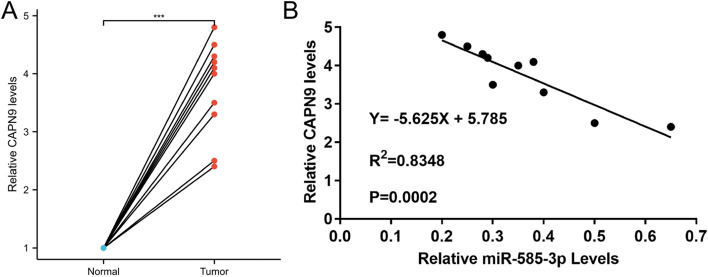
The expression of CAPN9 in HGSOC tissues and correlation between CAPN9 and miR-585-3p. **A** CAPN9 levels in 10 HGSOC tissues and their matched adjacent normal tissues were assessed via qRT-PCR. Relative CAPN9 level tumor/normal (log2) is listed. **B** Pearson's correlation coefficient analysis revealed that miR-585-3p levels and CANP9 mRNA levels were inversely correlated in the HGSOC tissue samples

**Fig. 7:**
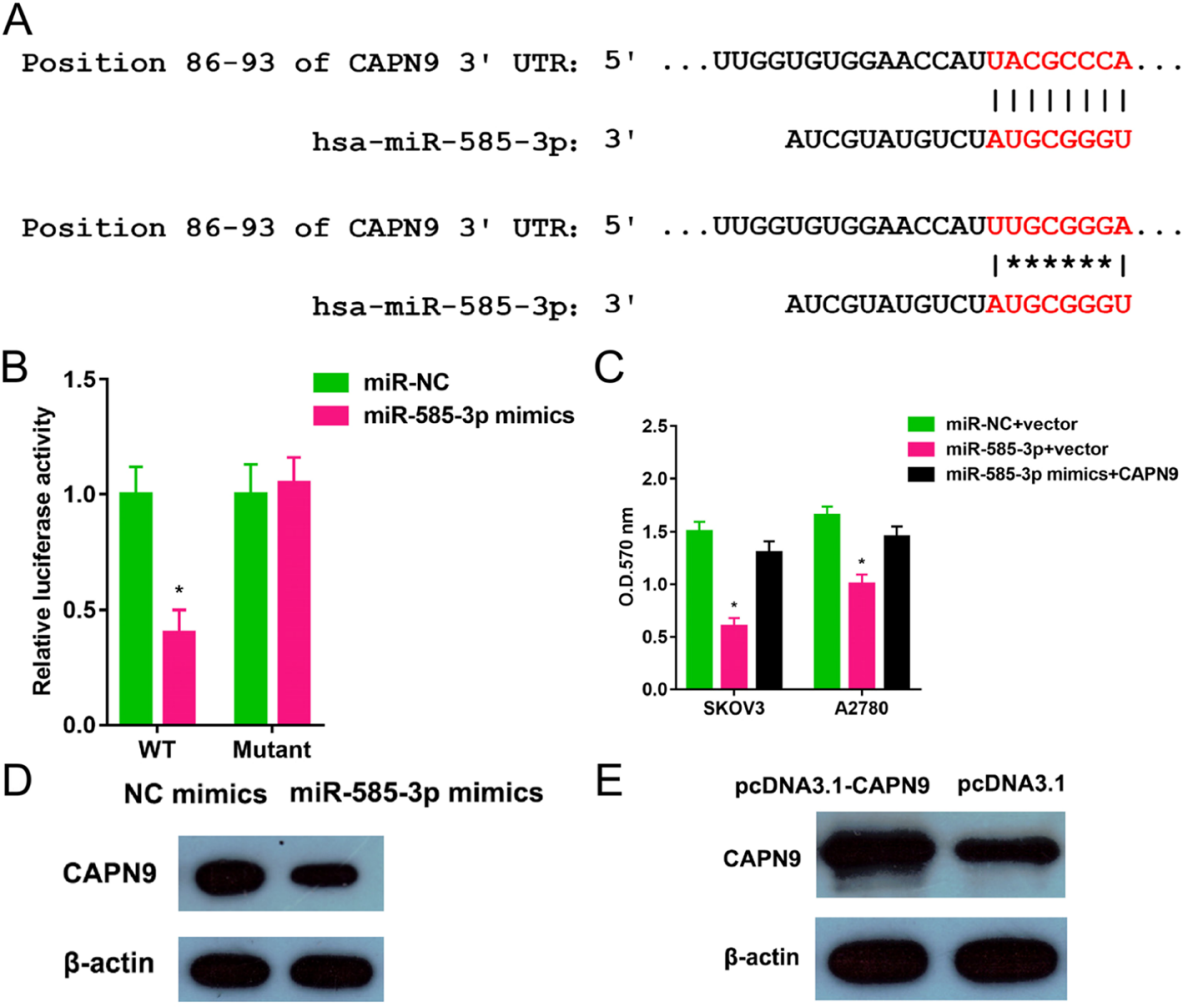
miR-585-3p targeted CAPN9. **A** Binding site locations (positions 86–93) were listed. **B** miR-585-3p mimics and a plasmid containing either a wild-type or mutated 3’-UTR sequences of CAPN9 were transferred into SKOV3 cells. **C** MTT assay was performed. *P < 0.05. **D** The amount of CAPN9 protein was determined via western blot. **E** The western blot was carried out to test the CAPN9 protein levels following transfection

As shown in Fig. 5, there were 24 common genes Common genes for miR-585-3p target genes predicted by TargetScan and miRDB. The common genes included C6orf136, HOGA1, MITF, CAPN9, CD34, RAVER2, VPS4A, CDC25B, GPR12, SPOCK1, VMAC, EXTL3, ATP6V1C1, ZFYVE26, PRPF4, GPRIN1, FSCN1, TMEM102, HAVCR1, DEDD, TRPV3, SMG1, SPCS3, and RNF213. The tumor tissues showed higher CAPN9 levels (Fig. 6A). CAPN9 expression was inversely correlated with miR-585-3p expression in HGSOC (Fig. 6B). As shown in Fig. 7A, the relationship between miR-585-3p and CAPN9 was investigated using sentinel mutations. In SKOV3 cells, miR-585-3p targeted CAPN9 (Fig. 7B). CAPN9 overexpression reduced the inhibitory effect of miR-585-3p on SKOV3 and A2780 cells (Fig. 7C). As shown in Fig. 7D, miR-585-3p transfection inhibited CAPN9 protein expression in SKOV3 cells. The results of the Western blot assay show the effect of transfection of pcDNA3.1-CANP9 in the SKOV3 cell line (Fig. 7E).

## Discussion

In this study, MiR-585-3p was significantly lower expressed in tumor tissues and cell lines than in normal tissues and cell lines. miR-585-3p overexpression inhibited cell growth and migration of OC cell lines. miR-585-3p downregulation promoted cell growth and migration of OC cell lines. These suggested that miR-585-3p played an inhibitory role in HGSOC.

In this study, bioinformatics analysis indicated that the CAPN9 gene is one of the miR-585-3p targets. The results of molecular biology experiments showed that CAPN9 was a novel downstream target of miR-585 and CAPN expression was regulated by miR-585 through direct binding to the 3'-UTR.

Calpains, a family of calcium-dependent cysteine proteases, consist of more than ten genes and contain two classes: classical calpains and non-classical calpains [17]. Calpains were associated with cellular processes, such as apoptosis and migration [18, 19]. Calpain-9 (encoded by CAPN9) (also known as nCL-4) is a more recently characterized member of the colpain family that was originally thought to be expressed in a digestive tract tissue-dependent manner. Gene expression of CAPN9 has turned out to be lowered in gastric cancer [20]. CANP9 is reflected in invasive breast cancer and is not expressed solely in a digestive tract specific manner [21]. In this study, CAPN9 was expressed at significantly higher levels in tumor tissues and cell lines than in normal ovarian tissue cell lines. The consequence was consisted with the result (Tumor: 1.58, Normal: 0.13) from GEPIA database (http://gepia.cancer-pku.cn/detail.php?GEPIA=GEPIA). We speculated that this may be due to different mechanisms of action mediated by the CAPN9 gene in different tumors and in different stages of development of the same tumor.

Circular RNA hsa_circRNA_102958 regulates miR-585/CDC25B to promote tumorigenesis in colorectal cancer [22] (17). Lnc01436 inhibits miR-585-3p expression, upregulates MAPK1 expression, and promotes gastric cancer progression [23]. Lnc01436, an oncogenic lncRNA in gastric cancer, regulated miR-585 and FBOX11 and promoted proliferation and metastasis of GC cells [24]. LncRNA H19 regulates miR-585-3p/PIK3R3 to attenuate MPTP-induced apoptosis in Parkinson's disease [25]. The GAS6-AS1/miR-585/EIF5A2 pathway plays an important role in HCC progression and is a potential target for therapeutic approaches in HCC [26] (21). The relationship between miR-585/CANP9 and lncRNAs has yet to be investigated. miR-585-3p acted directly on the target gene CAPN9 to promote the proliferation and migration of HGSOC.

This study was unable to investigate the relationship between miR-585-3p expression and the clinical characteristics of HGSOC patients due to the limited number of HGSOC tumor tissue samples. The relationship between miR-585-3p expression and the prognosis of HGSOC patients needs to be further studied.

## Conclusion

MiR-585-3p was expressed in lower levels in ovarian cancer tissues and cell lines than in normal ovarian tissues and cell lines. Down-regulated miR-585 inhibited the growth and migration of HGSOC. miR-585-3p acted directly on the target gene CAPN9 to promote the proliferation and migration of HGSOC. Targeted treatment based on miR-585/CANP9 is beneficial to improve the therapeutic outcome of HGSOC patients.

## Supplementary Information

**Additional file 1: Table S1. **The clinical characteristics of the patients in this study.

**Additional file 2: Table S2**. The sequence of primers in the present study.

## Data Availability

All data generated or analyzed during this study are included in this published article.
