# Supplementary material for: MiR-585-3p suppresses tumor proliferation and migration by directly targeting CAPN9 in high grade serous ovarian cancer
Source: J Ovarian Res. 2021 Jul 8;14:90. doi: 10.1186/s13048-021-00841-w (PMC8268593; doi:10.1186/s13048-021-00841-w)
Supplement: Supplementary file 1 — Additional file 1: Table S1. The clinical characteristics of the patients in this study. [file 13048_2021_841_MOESM1_ESM.doc]

**Table S1. The clinical characteristics of the patients in this study.**

| **No.** | **Age** | **History** | **Stage** | **Grade** |
| --- | --- | --- | --- | --- |
| 1 | 56 | High grade Serous ovarian cancer | III | 3 |
| 2 | 58 | High grade Serous ovarian cancer | III | 3 |
| 3 | 64 | High grade Serous ovarian cancer | III | 3 |
| 4 | 62 | High grade Serous ovarian cancer | III | 3 |
| 5 | 65 | High grade Serous ovarian cancer | IV | 3 |
| 6 | 67 | High grade Serous ovarian cancer | IV | 3 |
| 7 | 61 | High grade Serous ovarian cancer | IV | 3 |
| 8 | 66 | High grade Serous ovarian cancer | IV | 3 |
| 9 | 63 | High grade Serous ovarian cancer | III | 3 |
| 10 | 64 | High grade Serous ovarian cancer | III | 3 |
