# Supplementary material for: MiR-585-3p suppresses tumor proliferation and migration by directly targeting CAPN9 in high grade serous ovarian cancer
Source: J Ovarian Res. 2021 Jul 8;14:90. doi: 10.1186/s13048-021-00841-w (PMC8268593; doi:10.1186/s13048-021-00841-w)
Supplement: Supplementary file 2 — Additional file 2: Table S2. The sequence of primers in the present study. [file 13048_2021_841_MOESM2_ESM.doc]

**Table S2. The sequence of primers in the present study.**

| **Gene** |  | **The sequence of primers (5’-3’)** |
| --- | --- | --- |
| miR-585-3p | Forward | ACGCGTTCTCCTTACCATCCCTGA |
|  | Reverse | CGATCTGGAAGTAACCCAAGCC |
| U6 | Forward | CTCGCTTCGGCAGCACA |
|  | Reverse | AACGCTTCACGAATTTGCGT |
| CAPN9  (NM_006615.3) | Forward | AGTGGCGTTCTGTTGGT |
|  | Reverse | GGCATCGGGAGTGAGGT |
| GAPDH | Forward | CGACAGTCAGCCGCATCTTC |
|  | Reverse | CGTTCTCAGCCTTGACGGTG |
| miR-585-3p mimics |  | TGGGCGTATCTGTATGCTA |
| miR-585-3p ASO |  | ACCCGCATTCTGTATGCTA |
